# Exceptional long-term survival in pulmonary large-cell neuroendocrine carcinoma with brain metastases: a case report

**DOI:** 10.3389/fonc.2026.1631889

**Published:** 2026-01-20

**Authors:** Bin Liu, Liangwen Zhang, Hua Wang

**Affiliations:** 1 Department of Neurosurgery, Shandong Provincial Hospital Affiliated to Shandong First Medical University, Jinan, China; 2 Department of Neurology, Shandong Provincial Hospital Affiliated to Shandong First Medical University, Jinan, China

**Keywords:** brain metastasis, large-cell neuroendocrine carcinoma, long-term survival, molecular profiling, multimodal therapy, PRO-GRP, RB1, SDHD

## Abstract

**Background:**

Large-cell neuroendocrine carcinoma (LCNEC) of the lung is a rare and aggressive malignancy, with a poor prognosis and limited therapeutic options, particularly in advanced stages with brain metastases. Long-term survival in metastatic LCNEC is exceedingly uncommon.

**Case presentation:**

We report a case of a 32-year-old female diagnosed with stage IV pulmonary LCNEC in 2015. Over a 10-year disease course, she underwent a comprehensive, multimodal treatment approach, including chemotherapy, targeted therapy, two craniotomies, whole-brain radiotherapy, and resection of pelvic metastases. Serial imaging revealed prolonged stability of pulmonary lesions and dynamic control of brain metastases. Longitudinal monitoring of pro-gastrin-releasing peptide (PRO-GRP) levels showed a strong correlation with tumor progression. Molecular profiling identified RB1 and SDHD copy number loss, among other alterations. The patient remains alive 115 months after diagnosis and over 50 months after initial brain metastasis, representing one of the longest documented survivals in metastatic LCNEC.

**Conclusion:**

This case demonstrates that prolonged survival in stage IV LCNEC with brain metastases is possible through individualized multimodal therapy and close longitudinal monitoring. It underscores the potential value of molecular profiling and biomarkers like PRO-GRP in treatment planning and disease tracking.

## Introduction

Large-cell neuroendocrine carcinoma (LCNEC) of the lung is a rare, high-grade neuroendocrine tumor that accounts for approximately 2% to 3% of all primary lung malignancies (1, 2). Despite being classified alongside small-cell lung cancer (SCLC) within the World Health Organization’s neuroendocrine tumor spectrum (3, 4), LCNEC exhibits a wide range of morphological, clinical, and molecular characteristics that span both SCLC and non-small cell lung cancer (NSCLC) phenotypes (5–7). Moreover, LCNEC exhibits inconsistent clinical behavior with pathological and molecular prognostic indicators. Differential diagnosis hinges on distinguishing it from SCLC (smaller cells, scant cytoplasm, inconspicuous nucleoli) and NSCLC via morphology and IHC (such as Scgn more sensitive than CgA/Syn). Broad molecular analysis reveals two subtypes: TP53+STK11/KEAP1-mutant (type I) and TP53+RB1-mutant (type II), clarifying its heterogeneous nature straddling SCLC/NSCLC.

Due to its rarity and histological heterogeneity, the optimal management of LCNEC remains controversial (8). While surgical resection is generally favored for early-stage disease, the role of systemic therapy in advanced or metastatic settings is less well defined. Chemotherapeutic regimens traditionally used for SCLC—particularly platinum plus etoposide—have demonstrated some efficacy in LCNEC, although response rates tend to be lower compared to those observed in SCLC (1, 2). Moreover, emerging data suggest that LCNEC comprises at least two distinct molecular subtypes: one that is genetically similar to SCLC, often harboring co-alterations in TP53 and RB1, and another that mirrors NSCLC-like profiles, frequently featuring mutations in STK11, KRAS, and KEAP1 (6, 7, 9). Receptor tyrosine kinases, including MET and PDGFR, may play a role in the pathogenesis and progression of LCNEC (10).

Recent genomic studies have further revealed that actionable alterations in LCNEC are not uncommon (11). Mutations in the PI3K/AKT/mTOR pathway, alterations in chromatin remodeling genes such as MEN1 and ARID1A, and gene amplifications involving TERT or MYC family members have all been documented (7). These findings underscore the potential for precision medicine approaches in this challenging tumor subtype.

Brain metastases occur in a substantial proportion of LCNEC patients, with some cohorts reporting rates approaching 50% at diagnosis or during disease progression (2, 12, 13). However, there is a paucity of data regarding the efficacy of neurosurgical resection or stereotactic radiosurgery in these patients, and long-term survivors are exceedingly rare in published literature.

Here, we present a uniquely long-surviving case of metastatic LCNEC with extensive multi-organ involvement, including intracranial and pelvic metastases, managed through a multidisciplinary approach integrating surgery, radiotherapy, chemotherapy, and targeted therapy. This case illustrates not only the complex clinical behavior of LCNEC but also the potential for long-term survival with personalized multimodal treatment strategies. The case is further enriched by detailed radiological follow-up, longitudinal tumor marker data, and molecular profiling, offering valuable insights into disease progression and therapeutic response.

## Methods

Clinical, radiological, pathological, and molecular data were retrospectively collected from a single patient diagnosed with stage IV pulmonary LCNEC. Radiological assessments included serial brain MRI and chest/abdominal CT scans, which were reviewed by two independent radiologists. Tumor measurements were based on the largest diameter per lesion.

Histopathological and immunohistochemical evaluations were performed on both the primary and metastatic lesions using formalin-fixed, paraffin-embedded (FFPE) tissues. The neuroendocrine phenotype was confirmed by positive staining for chromogranin A (CgA), synaptophysin (Syn), and CD56. Ki-67 proliferation index was evaluated at different stages of disease progression.

Comprehensive molecular profiling was conducted via next-generation sequencing (NGS) on resected tissue specimens. Key genomic alterations—including RB1 and SDHD copy number losses and other fusion genes-were identified and correlated with disease behavior (14).

PRO-GRP serum levels were longitudinally monitored to evaluate neuroendocrine tumor activity. All procedures were performed with patient consent and approved by the institutional review board.

## Case presentation

**Figure 1:**
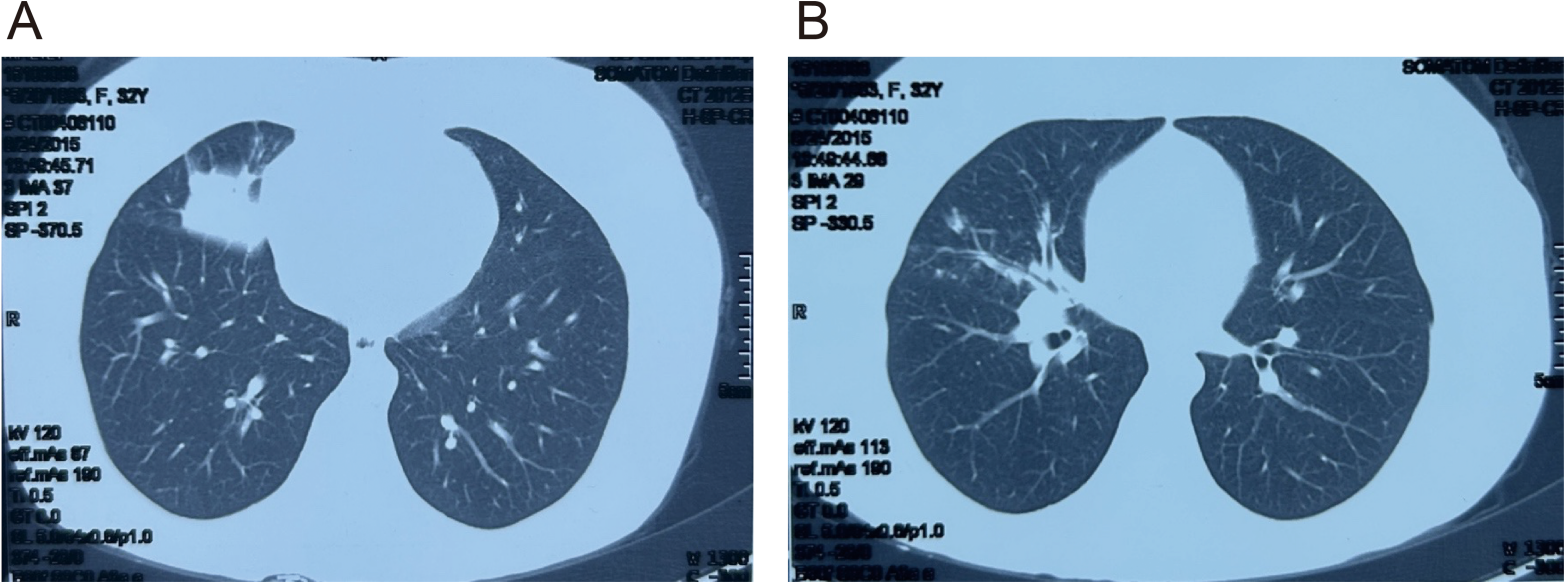
Chest CT images showing lesions in the right middle lobe and right hilum (September 24, 2015). **(A)** The CT scan revealed an irregular nodular opacity with ground-glass and high-density patches in the right middle lobe, with partial consolidation. The segmental bronchi remained patent. **(B)** The lesion measured approximately 4.0 × 4.0 cm. In the right hilum, an enlarged irregular soft tissue mass adjacent to the bronchus was seen, measuring 3.9 × 2.0 cm, with mild bronchial compression.

A 32-year-old female non-smoker was first diagnosed with pulmonary large-cell neuroendocrine carcinoma (LCNEC) in September 2015 following evaluation for persistent cough and dyspnea. Chest computed tomography (CT) revealed two primary lesions in the right lung: an irregular nodular mass measuring 4.0 × 4.0 cm in the right middle lobe and a 3.9 × 2.0 cm soft tissue lesion adjacent to the right hilum (**Figure 1**). Histopathological analysis confirmed LCNEC with neuroendocrine features and a Ki-67 index of 30%.

**Figure 2:**
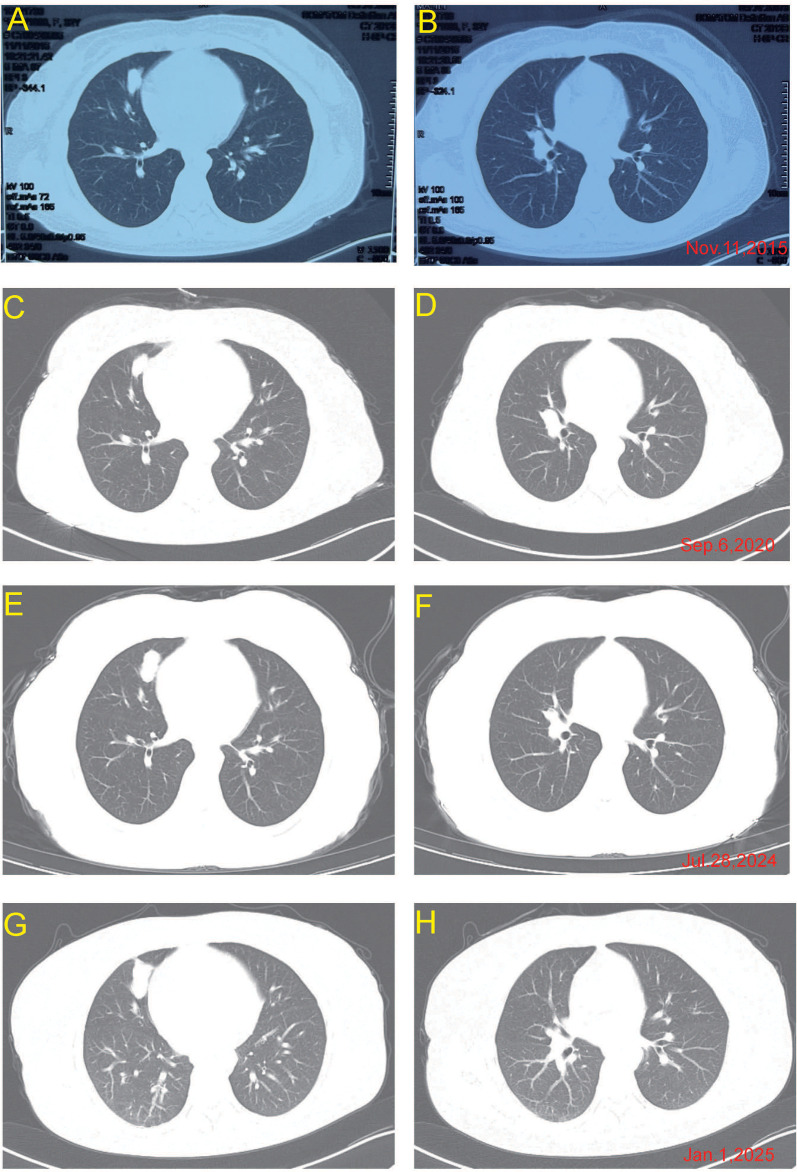
Chest CT follow-up from 2015 to 2025 showing changes in the right middle lobe and hilar lesions. Serial CT images from multiple timepoints demonstrate dynamic changes in the pulmonary lesions over a 10-year period, including post-chemotherapy regression, stable disease, and eventual progression in early 2025. The panels represent: **(A, B)** Nov 11, 2015; **(C, D)** Sep 6, 2020; **(E, F)** Jul 28, 2024; **(G, H)** Jan 4, 2025.

From 2015 to 2020, the patient underwent multiple cycles of chemotherapy including platinum-based doublets and achieved a prolonged period of disease stability. Serial imaging over these years revealed no evidence of distant metastases, and the primary lung lesions remained largely unchanged (**Figure 2**).

In September 2020, the patient presented with new-onset seizures. Brain magnetic resonance imaging (MRI) revealed multiple enhancing intracranial lesions, the largest of which was located in the right temporoparietal region, consistent with brain metastases. The patient underwent her first craniotomy on September 10, 2020. Postoperative pathology confirmed metastatic LCNEC, and immunohistochemistry remained positive for synaptophysin, chromogranin A, and CD56, with Ki-67 increasing to 70% (**Supplementary Figure 2**).

**Figure 3:**
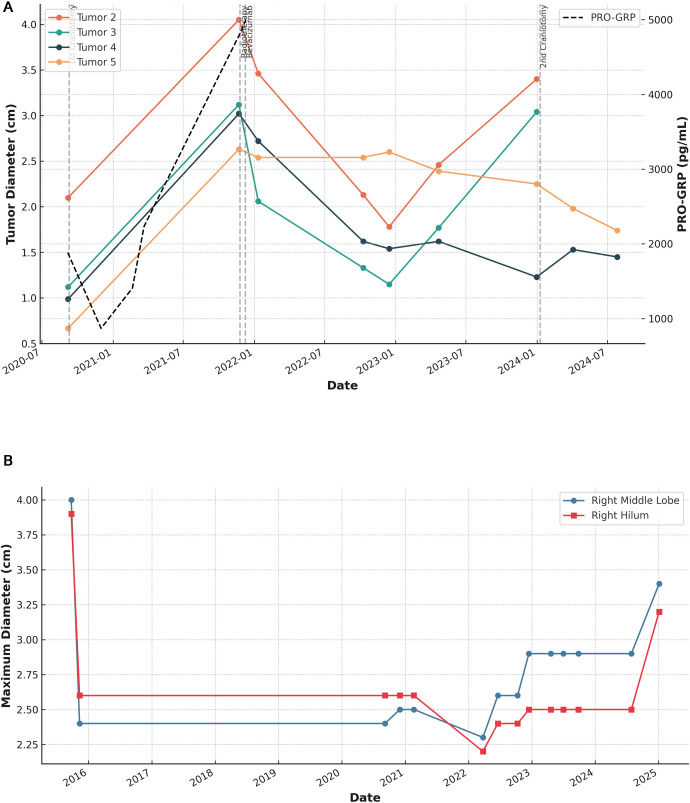
**(A)** Longitudinal changes in brain metastasis diameters and PRO-GRP. The temporal changes in the maximum diameters of four intracranial metastatic lesions (Tumor 2–5) from 2020 to 2024. A dashed black line represents serum PRO-GRP levels plotted on the secondary (right) y-axis. Vertical dashed lines indicate major therapeutic interventions: First craniotomy (September 10, 2020); Radiotherapy (November 25, 2021); Bevacizumab administration (December 9, 2021); Second craniotomy (January 9, 2024). **(B)** Changes in pulmonary lesions over time. The longitudinal changes in the maximum diameters of lesions in the right middle lobe and right hilum from 2015 to 2025. The trend demonstrates a long period of disease stability followed by notable progression in early 2025.

Following surgery, she received whole-brain radiotherapy and began systemic treatment with bevacizumab in combination with chemotherapy. A second course of brain radiotherapy was administered in late 2021. Longitudinal follow-up with serial brain MRI showed dynamic changes in the four largest metastatic lesions (Tumors 2–5), including partial responses and later progression (**Supplementary Figure 1**, **Figure 3A**).

In December 2023, a second craniotomy was performed due to disease progression in the bilateral frontal lobes. Postoperative imaging revealed satisfactory resection and a small postoperative subdural empyema, which resolved with antibiotics (**Supplementary Figure 3**).

In parallel with brain disease management, abdominal imaging in 2021 revealed a solitary pelvic mass, which was surgically removed and confirmed as metastatic LCNEC. Additionally, a progressive increase in the size of the right pulmonary lesions was noted in early 2025 (**Figure 3B**), marking the end of a nearly 10-year period of pulmonary stability.

**Figure 4:**
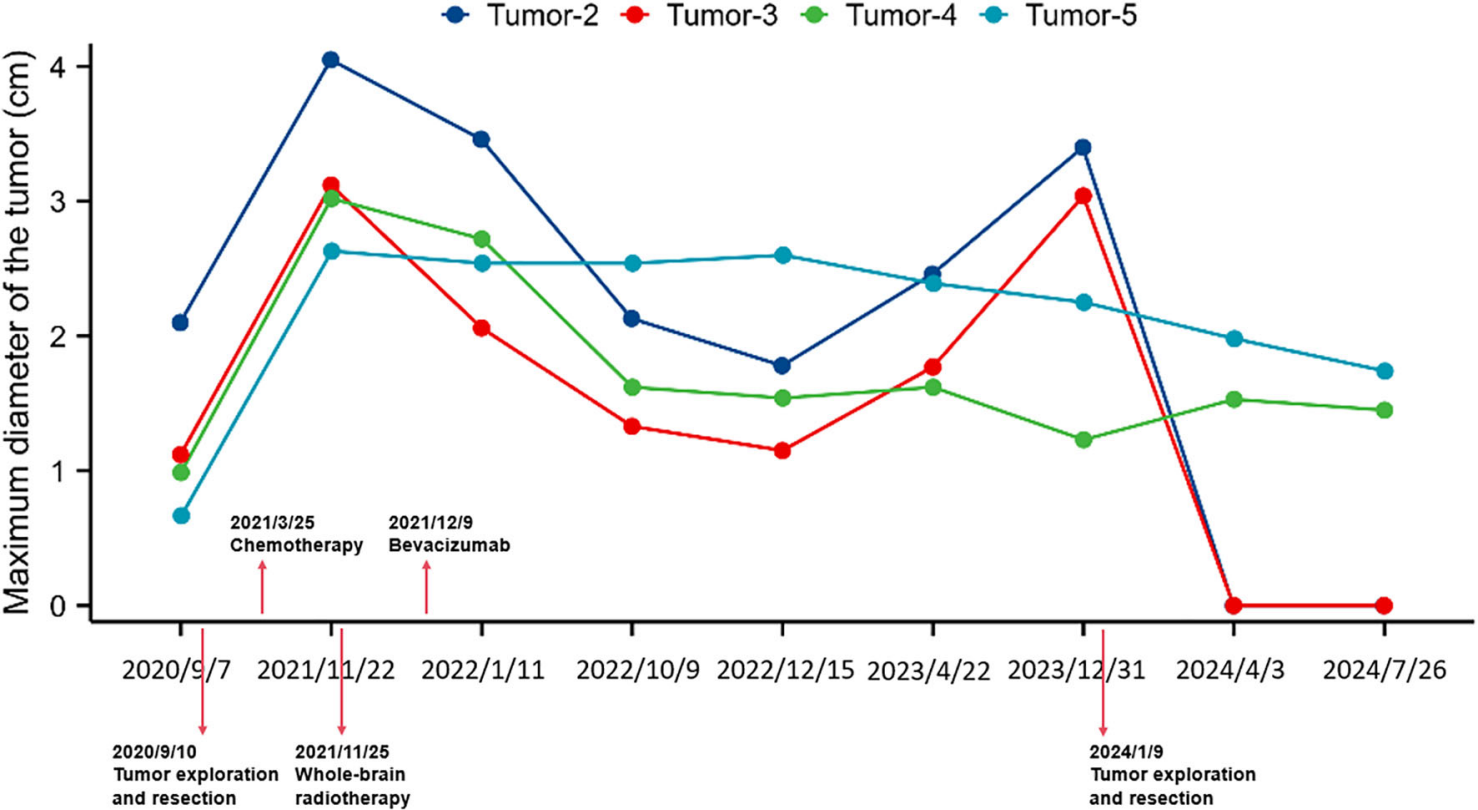
Changes in the maximum diameter of the tumor and corresponding interventions since September 7, 2020.

Serial assessments of the serum biomarker pro-gastrin-releasing peptide (PRO-GRP) showed a marked elevation during disease progression phases, particularly around intracranial and pelvic metastases, suggesting a correlation with tumor burden (**Figure 3A**). **Figure 4** illustrates the changes in the maximum diameter of the four intracerebral metastatic tumors in this patient since September 7, 2020, and further shows that the patient underwent exploratory resection of the right temporoparietal tumor on September 10, 2020. On March 25, 2021, the patient received one cycle of chemotherapy with the regimen: carboplatin 400mg d1 + etoposide 0.2g d2. Whole-brain radiotherapy was initiated on November 25, 2021, with a single dose of 300 cGy, administered for a total of 10 fractions. On December 9, 2021, bevacizumab was administered for anti-angiogenic therapy; it was used as a single agent once at a dose of 600 mg via intravenous drip stat. A follow-up examination on December 31, 2023, revealed significant enlargement of two of the tumor lesions, and the patient underwent exploratory resection of the bilateral frontal lobe tumors on January 9, 2024.

To date, the patient has survived more than 115 months since initial diagnosis and over 50 months since brain metastasis, making this one of the longest reported survival durations in stage IV LCNEC with extensive intracranial and extracranial metastases.

## Discussion

This case highlights the remarkable long-term survival of a patient with metastatic LCNEC, a cancer subtype typically associated with dismal prognosis and aggressive clinical course. The median overall survival (OS) for advanced LCNEC is generally reported to be less than 12 months, particularly in cases with brain metastases (2, 13). Our patient, however, has survived over 9 years since initial diagnosis and more than 4 years post-brain metastasis, placing her among the longest-surviving LCNEC patients reported in the literature—a outcome that underscores the potential variability in disease behavior within this heterogeneous subtype.

Several factors may have contributed to this exceptional individual outcome, though their broader applicability requires careful consideration given the rarity of such cases. First, the patient benefited from a personalized, multimodal therapeutic approach that included early neurosurgical resection (15), serial radiotherapy, multiple lines of chemotherapy, targeted anti-angiogenic therapy (bevacizumab), and timely surgical management of pelvic metastasis (16). Notably, this patient achieved prolonged systemic disease stability with chemotherapy alone during the initial disease phase (2015-2020): after receiving multiple cycles of platinum-based doublet chemotherapy, serial chest/abdominal CT scans confirmed no evidence of distant extracranial recurrence for nearly 5 years, and the primary pulmonary lesions remained largely unchanged—this finding is striking, as it contrasts with the typical pattern of advanced LCNEC, which often progresses rapidly within 1–2 years of initial chemotherapy with systemic recurrence as the main cause of treatment failure. Gamma Knife or surgical intervention for brain metastases from LCNEC remains rare, and while this patient experienced prolonged survival following aggressive local control, such an approach may not be suitable for all individuals with metastatic LCNEC (13). Importantly, the patient’s first distant progression event in September 2020 was exclusively intracranial metastases (confirmed by whole-body CT and MRI, with no extracranial involvement), which aligns with the emerging clinical entity of “brain-only disease”—a phenomenon well-documented in NSCLC and SCLC but rarely reported in LCNEC, and one that merits attention for its unique management implications. Patient selection—including factors such as tumor burden, performance status, and the presence of extracranial disease—likely plays a critical role in determining which patients may derive benefit from intensive local therapy, and further data are needed to define these selection criteria.

Second, the molecular profile of this patient may have conferred a relatively indolent disease biology. Loss of RB1 and SDHD was observed in the metastatic lesions (17). RB1 inactivation is a defining feature of high-grade neuroendocrine tumors and is often associated with poor prognosis (9). However, some reports suggest that when RB1 loss coexists with other favorable features (such as low TMB, absence of TP53 mutation), a subset of LCNEC may display prolonged responses to cytotoxic agents (6, 7). The RB1 and SDHD copy number loss identified in this case may be potentially relevant to the observed “brain-tropic” metastatic pattern: RB1 inactivation (a hallmark of SCLC-like LCNEC) is linked to enhanced tumor cell penetration of the blood-brain barrier, while emerging evidence suggests an association between SDHD loss and metabolic reprogramming in neuroendocrine tumors—a phenomenon that might indirectly support tumor cell adaptation and survival in the unique intracranial microenvironment. It is important to note that no direct literature evidence currently supports a causal relationship between SDHD loss and brain tropism in LCNEC, and this metastatic pattern is more likely multifactorial, with RB1 inactivation and other uncharacterized molecular or microenvironmental factors potentially contributing synergistically. This molecular context may partially explain why the patient developed isolated brain metastases rather than systemic recurrence initially, though definitive causal links require further validation in larger LCNEC cohorts. Additionally, SDHD loss has been implicated in activation of the hypoxia-inducible factor (HIF) pathway and increased angiogenesis in neuroendocrine derivatives, which may provide a mechanistic rationale for the observed clinical response to bevacizumab (anti-angiogenic therapy) in this case. It is important to note that this molecular pattern is not universal in LCNEC, and the prognostic and therapeutic implications of RB1/SDHD co-alterations remain incompletely characterized in larger cohorts.

Notably, large cell neuroendocrine carcinoma (LCNEC) and small cell lung carcinoma (SCLC) are classified as high-grade pulmonary neuroendocrine tumors but exhibit distinct specificity in histomorphology, molecular profiles, immunophenotype, and clinical behavior. Histopathologically, LCNEC is characterized by larger tumor cells with abundant cytoplasm, prominent nucleoli, and a lower nuclear-cytoplasmic ratio, whereas SCLC presents with small fusiform cells, scant cytoplasm, and dense chromatin without distinct nucleoli. At the molecular level, SCLC is typically defined by universal TP53/RB1 co-inactivation and subtype-specific drivers (e.g., ASCL1, NEUROD1), while LCNEC displays marked heterogeneity, being categorized into SCLC-like (RB1/TP53 deletion) and NSCLC-like (KRAS/STK11 mutation) subtypes with a lower RB1 mutation frequency (26% vs. 40% in SCLC) and unique alterations in the PI3K/AKT/mTOR pathway. Immunophenotypically, LCNEC shows higher expression of CK7, CK18, and E-cadherin compared to SCLC, along with lower TTF-1 positivity and a Ki-67 index predominantly ranging from 50% to 80% (vs. 80%-100% in SCLC). Clinically, despite similar overall survival, LCNEC exhibits poorer response to platinum-based chemotherapy (overall response rate ~30%-35% vs. ~56%-61% in SCLC) and a greater role for surgical resection in resectable stages. These distinctive features highlight the necessity of integrating histopathological examination, immunophenotypic profiling, and molecular testing for accurate differentiation, which is crucial for guiding individualized therapeutic strategies.

Third, the patient’s prolonged pulmonary stability (nearly 10 years) is notable. Lung lesions remained stable for years before eventual progression, suggesting early therapeutic response and effective immune surveillance. The rise in Ki-67 from 30% to 70% during progression also underscores the dynamic nature of LCNEC biology.

Furthermore, the patient’s PRO-GRP levels correlated well with disease activity, and may represent a useful biomarker for monitoring tumor burden in LCNEC, analogous to its use in SCLC (18). While not routinely employed in LCNEC, our findings support further exploration of PRO-GRP as a real-time disease activity indicator (18). Notably, compared to recently reported prognostic models and biomarkers, PRO-GRP exhibits distinct advantages: its serum-based detection is widely accessible, cost-effective (avoiding the high expenses of sequencing or multi-assay panels), and enables real-time tracking of tumor dynamics without repeated invasive tissue biopsies—addressing unmet needs for routine surveillance in LCNEC (19–22).

Finally, this case emphasizes the clinical value of longitudinal radiographic and molecular monitoring. Radiological follow-up using MRI and CT allowed early detection and precise tracking of intracranial and pulmonary changes (**Supplementary Figure 1**, **Figure 3**), while molecular profiling guided therapeutic decision-making. Given the patient’s presentation of brain-only disease, these findings also suggest that regular brain MRI surveillance (even in the absence of neurological symptoms) should be considered for LCNEC patients who achieve initial systemic stability with chemotherapy, to enable early detection and intervention for isolated brain metastases. Such data-rich follow-up is rarely seen in LCNEC literature (23).

In conclusion, this case demonstrates that long-term survival is possible in select patients with LCNEC and brain/distant metastases when treated with an aggressive, individualized multimodal strategy. Personalized multimodal therapy, meticulous follow-up, and integration of molecular diagnostics may play important roles in optimizing outcomes for such patients, though their generalizability across diverse LCNEC populations requires further investigation. A dual-path classification system may offer value for guiding treatment in advanced-stage LCNEC, but its clinical utility also needs validation in prospective trials (24). Notably, this case also highlights the potential for long-term systemic control with initial chemotherapy alone in LCNEC, as well as the existence of brain-only disease as a distinct progression pattern—two observations that warrant further investigation to refine surveillance and treatment strategies for this rare malignancy. Given the rarity and high heterogeneity of LCNEC, this single case cannot be used to establish a universal clinical guideline for all patients with metastatic LCNEC and brain metastases. The therapeutic approach employed here was tailored to the unique clinical, radiological, and molecular features of this specific patient, and its applicability to other individuals may be limited by variations in tumor biology, patient comorbidities, and access to multimodal care. Future patients with metastatic LCNEC should undergo comprehensive, individualized assessment—including molecular profiling, radiological staging, and evaluation of functional status—before determining a treatment strategy. Further large-scale, prospective studies are warranted to better characterize the prognostic and therapeutic implications of LCNEC molecular subtypes, define optimal patient selection for aggressive local therapy (such as neurosurgical resection for brain metastases), and validate biomarkers like PRO-GRP for broader clinical use.

The patient provided written informed consent for publication of all clinical, radiological, and molecular data included in this report.

## Data Availability

The original contributions presented in the study are included in the article/**Supplementary Material**. Further inquiries can be directed to the corresponding authors.

## Supplementary material

Supplementary Figure 1 Serial MRI scans of brain metastases (Tumors 2–5) from Sep 2020 to Jul 2024. T1-weighted contrast-enhanced MRI images show the evolution of four individual metastatic brain lesions across 10 timepoints. The images correspond to Tumors 2–5 from top to bottom, with scan dates from left to right as: Sep 7, 2020; Nov 22, 2021; Jan 11, 2022; Oct 9, 2022; Dec 15, 2022; Apr 22, 2023; Dec 31, 2023; Apr 3, 2024; Jul 26, 2024. The sequential changes reflect partial response and later progression.

Supplementary Figure 2 MRI before and after the first craniotomy. **(A)** Preoperative contrast-enhanced MRI on Sep 7, 2020 showed a large enhancing lesion in the right temporoparietal region with central necrosis and midline shift. **(B)** Postoperative FLAIR MRI on Feb 22, 2021 demonstrated significant reduction of the lesion and resolution of mass effect.

Supplementary Figure 3 MRI before and after the second craniotomy. **(A, B)** Preoperative contrast-enhanced MRI on Dec 30, 2023 revealed bilateral frontal lobe lesions with perilesional edema. **(C)** Postoperative MRI on Apr 3, 2024 showed residual cavity and the development of subdural empyema beneath the frontal scalp flap.
