# Supplementary figures and images for: Exceptional long-term survival in pulmonary large-cell neuroendocrine carcinoma with brain metastases: a case report
Source: Front Oncol. 2026 Jan 20;16:1631889. doi: 10.3389/fonc.2026.1631889 (PMC12864098; doi:10.3389/fonc.2026.1631889)

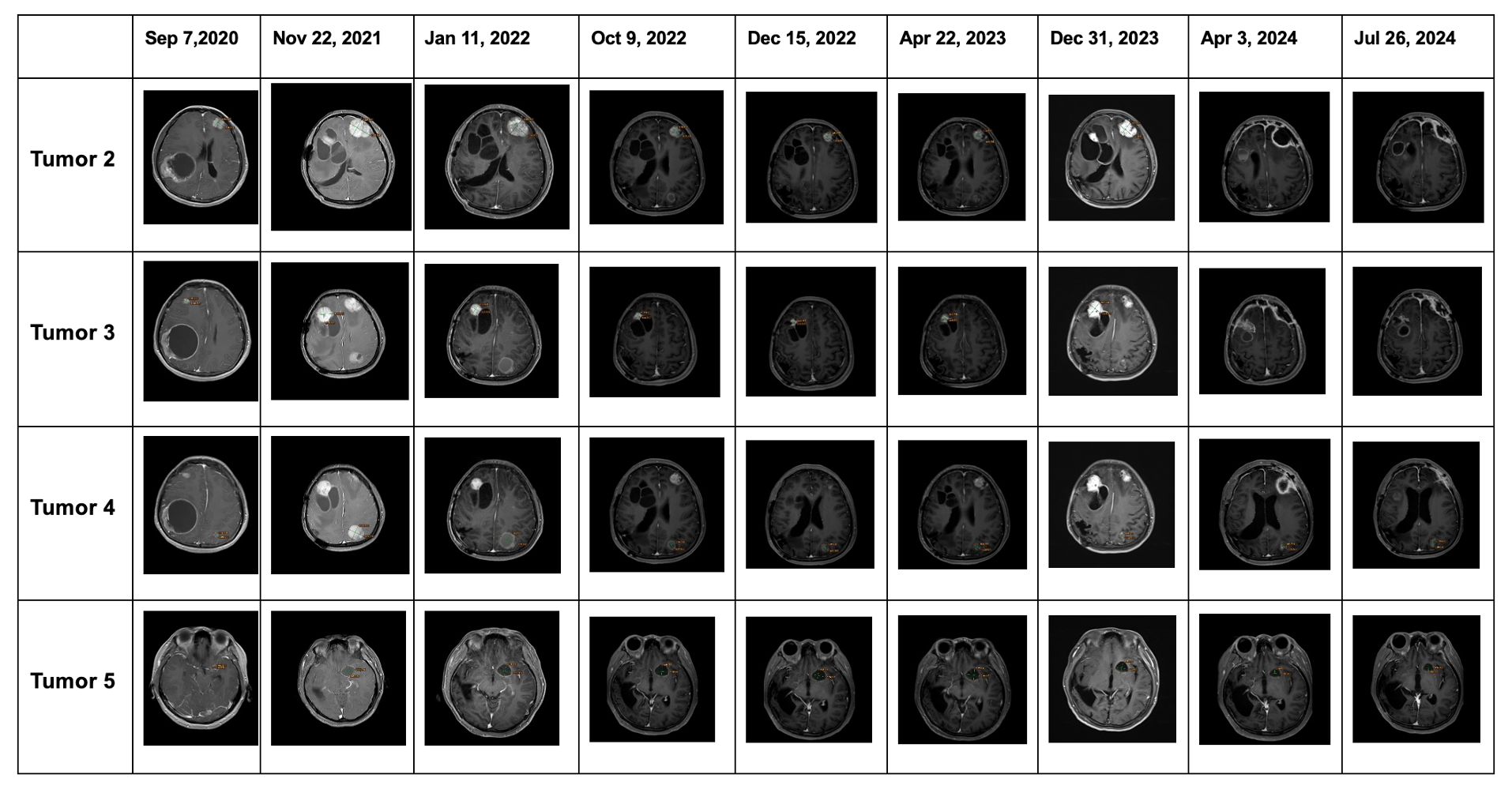

Supplement: Supplementary Figure 1 — Serial MRI scans of brain metastases (Tumors 2–5) from Sep 2020 to Jul 2024. T1-weighted contrast-enhanced MRI images show the evolution of four individual metastatic brain lesions across 10 timepoints. The images correspond to Tumors 2–5 from top to bottom, with scan dates from left to right as: Sep 7, 2020; Nov 22, 2021; Jan 11, 2022; Oct 9, 2022; Dec 15, 2022; Apr 22, 2023; Dec 31, 2023; Apr 3, 2024; Jul 26, 2024. The sequential changes reflect partial response and later progression. [file Image1.jpeg]

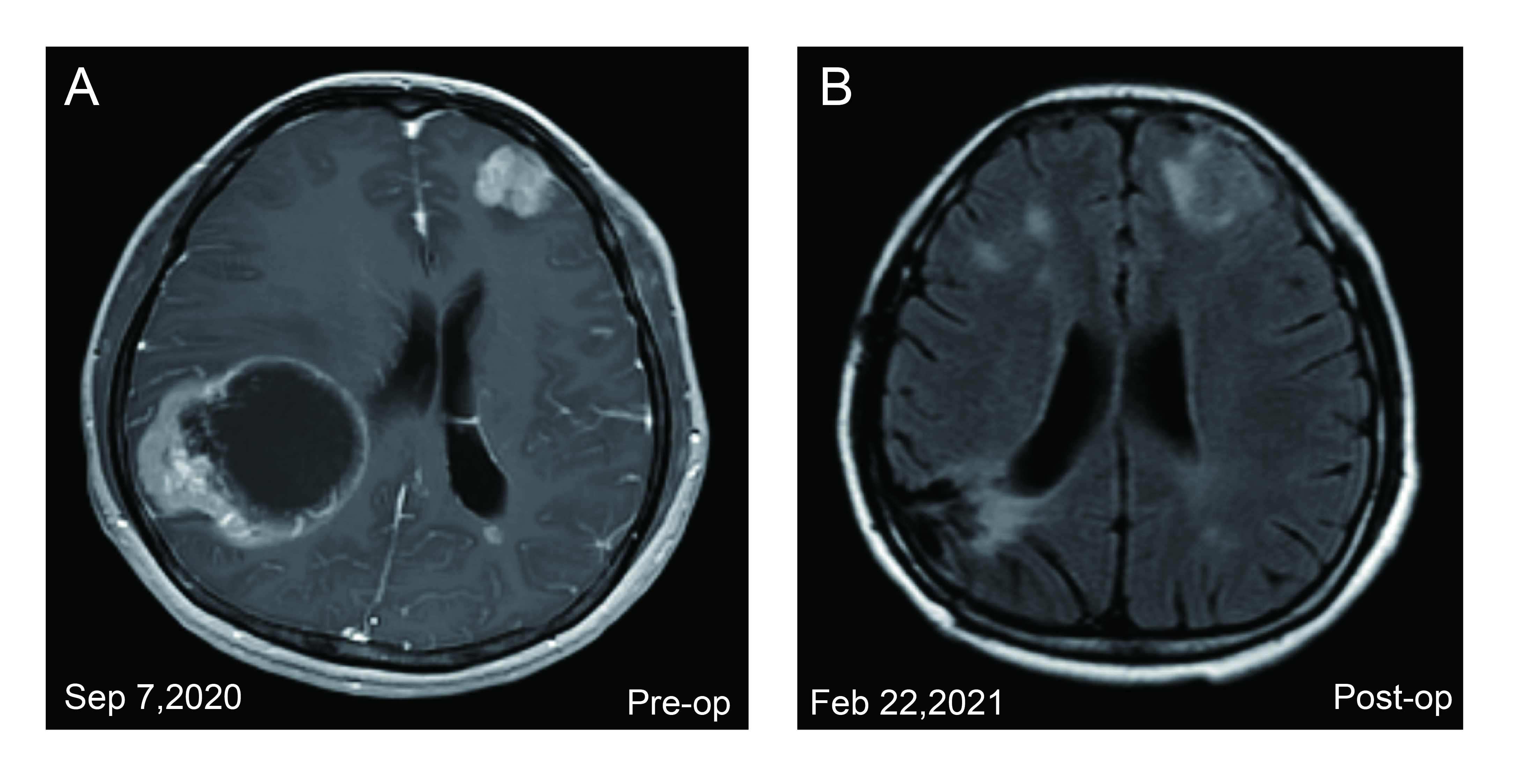

Supplement: Supplementary Figure 2 — MRI before and after the first craniotomy. (A) Preoperative contrast-enhanced MRI on Sep 7, 2020 showed a large enhancing lesion in the right temporoparietal region with central necrosis and midline shift. (B) Postoperative FLAIR MRI on Feb 22, 2021 demonstrated significant reduction of the lesion and resolution of mass effect. [file Image2.jpeg]

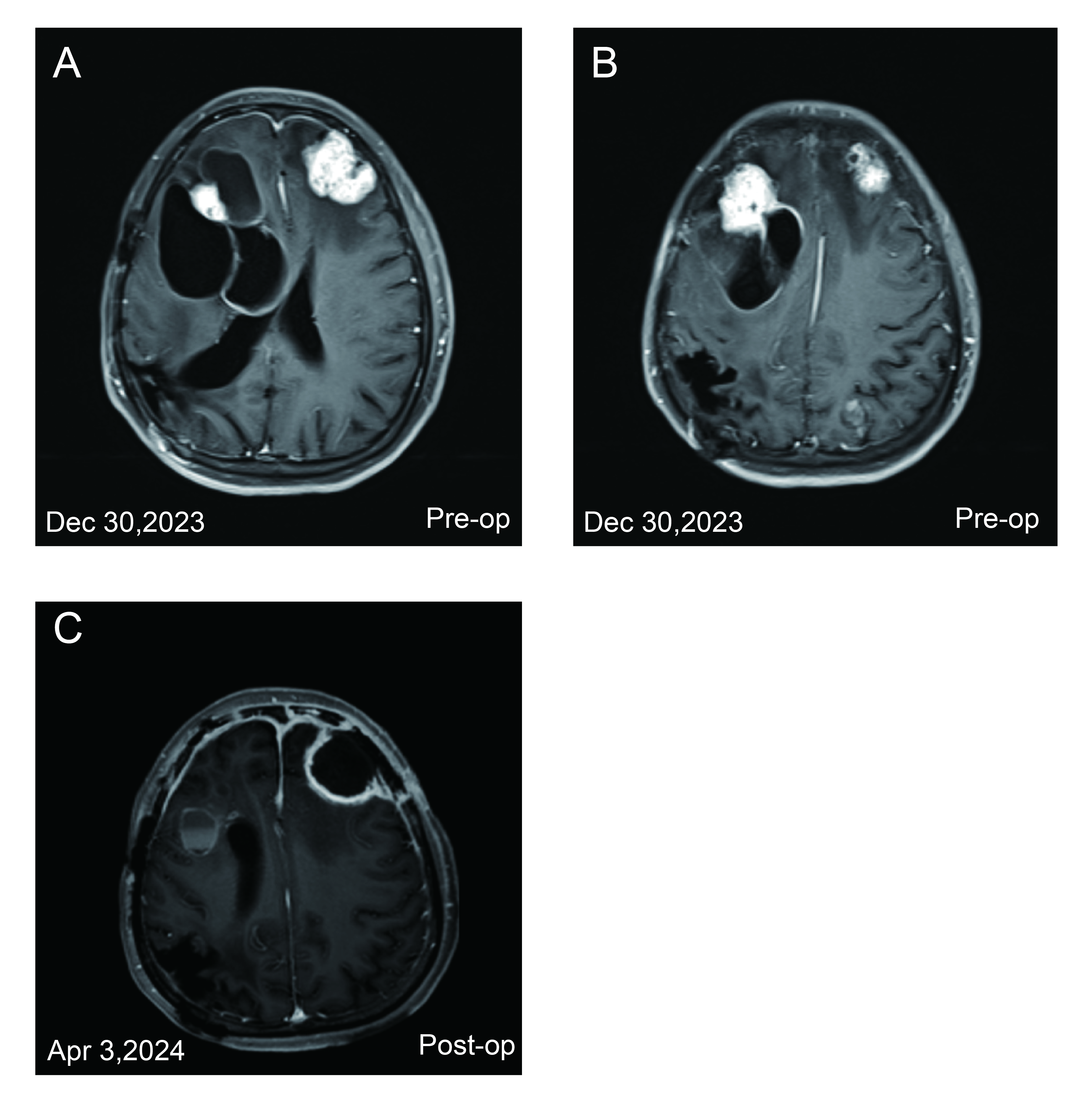

Supplement: Supplementary Figure 3 — MRI before and after the second craniotomy. (A, B) Preoperative contrast-enhanced MRI on Dec 30, 2023 revealed bilateral frontal lobe lesions with perilesional edema. (C) Postoperative MRI on Apr 3, 2024 showed residual cavity and the development of subdural empyema beneath the frontal scalp flap. [file Image3.jpeg]
